# Race, Neighborhood Economic Status, Income Inequality and Mortality

**DOI:** 10.1371/journal.pone.0154535

**Published:** 2016-05-12

**Authors:** Nicolle A Mode, Michele K Evans, Alan B Zonderman

**Affiliations:** National Institute on Aging, National Institutes of Health, Department of Health and Human Services, Baltimore, Maryland, United States of America; New York City Department of Health and Mental Hygiene, UNITED STATES

## Abstract

Mortality rates in the United States vary based on race, individual economic status and neighborhood. Correlations among these variables in most urban areas have limited what conclusions can be drawn from existing research. Our study employs a unique factorial design of race, sex, age and individual poverty status, measuring time to death as an objective measure of health, and including both neighborhood economic status and income inequality for a sample of middle-aged urban-dwelling adults (N = 3675). At enrollment, African American and White participants lived in 46 unique census tracts in Baltimore, Maryland, which varied in neighborhood economic status and degree of income inequality. A Cox regression model for 9-year mortality identified a three-way interaction among sex, race and individual poverty status (p = 0.03), with African American men living below poverty having the highest mortality. Neighborhood economic status, whether measured by a composite index or simply median household income, was negatively associated with overall mortality (p<0.001). Neighborhood income inequality was associated with mortality through an interaction with individual poverty status (p = 0.04). While racial and economic disparities in mortality are well known, this study suggests that several social conditions associated with health may unequally affect African American men in poverty in the United States. Beyond these individual factors are the influences of neighborhood economic status and income inequality, which may be affected by a history of residential segregation. The significant association of neighborhood economic status and income inequality with mortality beyond the synergistic combination of sex, race and individual poverty status suggests the long-term importance of small area influence on overall mortality.

## Introduction

Mortality disparities across racial and economic groups in the United States (US) are well established [1]. In 1995, African Americans had a 1.6 times greater overall mortality risk than Whites; unchanged from the mortality disparity observed in 1950 [2]. Low socioeconomic status (SES) is also associated with an increased mortality risk for the US population. For adults over age 50, those in the lowest quartile of SES had 2.8 times the mortality risk as those in the highest quartile of SES [3], and this disparity remained significant after controlling for major risk factors (1.6 times). The influence of race and SES on mortality are difficult to parse because African Americans bear a disproportionate burden of US poverty and low education. The poverty rate for African Americans in the US is 26%, but it is only 10% for non-Hispanic Whites [4]. Similarly, 15% of African Americans have less than a high school education, while 8% of non-Hispanic Whites fall in this category [5].

The influence of economic status on overall health and mortality extends beyond the individual to the neighborhood [6]. Place of residence in the US follows patterns of race and economic position, often due to residential segregation [7]. While racial segregation has decreased over the last 40 years [8], income segregation, especially for African Americans, has increased [9]. Low neighborhood economic status has been associated with an increased risk of overall mortality [10], and mortality from cancer [11] and cardiovascular disease [12]. Residing in neighborhoods with the lowest economic status (lowest 20 or 25th percentile) corresponded with a 17–26% increased risk of overall mortality after controlling for individual SES and disease risk factors [13, 14]. The influence of neighborhood can be direct, through walkability or violent crime, or indirect, through social position or discrimination. Due to the complex ways in which neighborhood can influence health, researchers have proposed composite indices which include multiple aspects of the neighborhood milieu (e.g., [15, 16]). However, researchers have found similar patterns between neighborhood economic status and health using only a single measure of poverty or median household income [17, 18]. Recently, Oka [19] demonstrated that median household income alone accounted for the same neighborhood affluence-deprivation continuum as composite measures for four large US cities at the census tract level.

In addition to average economic levels, neighborhood influences also include income disparities within neighborhoods. A number of studies have linked high income inequality with an increase in adverse health outcomes such as overall mortality [20, 21]. This has led some authors to posit that the criteria of causal association between income inequality and health has been reached [21]. The relative income hypothesis asserts that chronic upward comparisons are stressful [22] and adverse health outcomes are the result of the physical effects of chronic stress and social sensitivity [23]. Furthermore, research has identified an interaction between neighborhood economic status and income inequality on health. Two studies from California found that mortality risks for residents with low-incomes were highest in high SES neighborhoods [24, 25]. This association between income inequality and health is not universally supported [26]. Quality housing, access to healthy food, effective schools and a safe environment available in a high economic status neighborhood should benefit low income residents in the same area. These potential benefits have provided the foundation for projects promoting the relocation of low-income families such as Moving to Opportunity, which was conducted in five major cities across the US [27]. The project demonstrated limited success in reducing mortality risk factors for low-income residents who moved to more affluent neighborhoods [28].

Many studies of race, individual poverty and neighborhood economic status on mortality are limited in their conclusions due to existing correlations among these primary factors [12, 13]. Our study adds to previous work by employing a unique factorial design of race, sex, age and individual poverty status, measuring time to death as an objective measure of health, and including both neighborhood economic status and income inequality for a population of middle-aged urban-dwelling adults. The purpose of the current study was to identify significant individual and neighborhood components that correlate with mortality disparities. A secondary aim was to compare the explanatory power of neighborhood economic status by a composite index compared to median household income.

## Methods

### Study population

The Healthy Aging in Neighborhoods of Diversity across the Life Span (HANDLS) study is a prospective longitudinal cohort study of 3720 socioeconomically diverse African American and White adults initially 30–64 years old. Participants were selected using an area probability sample from thirteen local communities in Baltimore, Maryland, during 2004–2009. The local communities were chosen to span diverse levels of income and socioeconomic status and provide a representative distribution of Baltimore residents. Participants were 30–64 years old at enrollment, and selected based on a factorial crossed design of sex, race, 5-year age group, and poverty status (above/below 125% of the federal poverty guidelines based on household size). The factorial design allows analysis of the separate and combined associations of sex, race, and poverty status on health outcomes [29]. Participants were limited to those who self-identified as either non-Hispanic Black/African American or non-Hispanic White/Caucasian. Enrollment dates were similar for both races, with a median start date of August 2006 for African American participants and December 2006 for White participants. For this study, 43 participants were excluded who provided addresses that could not be geocoded accurately as were two participants who had permanent addresses just outside the Baltimore City limits, resulting in a study sample of 3675 people. Detailed descriptions of the protocol and methods have been previously published [30]. Approval for data collection was obtained from the National Institutes of Health, National Institute of Environmental Health Sciences Institutional Review Board. All participants provided written informed consent.

### Mortality information

Participants were followed prospectively via matching to National Death Index data (NDI; National Center for Health Statistics, Centers for Disease Control and Prevention). Individual data for matching included name, date and state of birth, sex, race, maiden name, and social security number. Minimal loss of follow-up was expected because 94% of the participants provided a social security number, and participants were actively contacted for follow-up visits throughout the study period. NDI data were available from the date of HANDLS enrollment (August 2004–March 2009) through December 31, 2013, providing up to 9 years of follow-up (mean and median of 6.9 years). Details included date of death and primary cause (International Classification of Disease 10th revision).

### Neighborhood-level information

The entire city of Baltimore, Maryland was included with census tracts used as small areas following the 2010 Census definitions. Census tracts include 4000 residents on average and are adequately sized for detecting spatial gradients and trends over time in overall mortality [17]. Data for each tract came from the American Community Survey (ACS) 5-year estimate files: 2006–2010 (referred to as 2010) and 2009–2013 (referred to as 2013). Nineteen variables previously identified as related to health outcomes were selected to cover seven domains of social condition and relative socioeconomic disadvantage: education, employment, housing, occupation, poverty, residential stability and financial security. These variables formed the list for possible inclusion in a neighborhood index (S1 Table). The Gini coefficient [31] from the ACS 2010 file was used as the measure of income inequality for each census tract. For this study, the percent Gini was used (Gini * 100) and thus values range from 0 (equal incomes) to 100 (all income held by one person). The percent Gini was used so model coefficients would describe the result of a 1% increase in Gini value.

### Index development: Neighborhood Economic Index (NEI)

The 19 selected neighborhood-level variables were included in a principal component analysis (PCA) to select a set of variables for the index (see S1 Appendix). Retained standardized variables were summed to create the index value without individual weights for greater consistency over time, with low values indicating low neighborhood economic level. Internal reliability was assessed by Cronbach's alpha with values greater than 0.90 indicating high reliability [32]. Polyserial correlations [33] of the neighborhood index with poverty status and education level assess the level of redundancy between the index and individual socioeconomic indicators.

### Statistical analyses

Cox proportional hazards models were used to estimate mortality hazard ratios (HR) and their Wald 95% confidence intervals (CI). Exact age at entry and exit of the study were used as the measurement of time for the models [34]. Enrollment in HANDLS was entry into the study and exit was date of death or December 31, 2013, whichever occurred first. Backward variable selection was performed using likelihood ratio tests to identify significant interactions and build the final model. Main effects of sex, race and poverty status were included *a priori* based on the design of the study and not removed during variable selection. Models were built separately using the NEI (Model 1) and neighborhood median income (Model 2), and Akaike information criterion (AIC) values were compared between the two resulting models. The assumption of proportionality was assessed by inspection and testing of the Schoenfeld residuals [35].

All analyses were performed in the R program [36] version 3.1.3 except for estimated interaction HRs which were calculated in SAS/STAT software version 13.2 (SAS Institute Inc., Cary NC). All p-values are two-tailed and values less than 0.05 were considered statistically significant.

## Results

### Index development: Neighborhood Economic Index (NEI)

Baltimore, Maryland has 200 census tracts and 198 of them contain at least one household. The PCA identified six variables for the index: percent of households with unemployed, percent of households with people out of the workforce, percent of households receiving food stamps, percent of households earning less than $30,000 annually, percent of households with no car and percent of households in poverty (details in S1 Appendix). NEI values were calculated as the sum of the six individually standardized variables, and varied from -16.6 to 10.3, with a median value of 0.5 (mean = 0). The six variables had high internal reliability, with a Cronbach's alpha value of 0.95, and the method demonstrated high repeatability when conducted on the ACS 2013 dataset (S1 Appendix). Correlation between the NEI and Gini Coefficient, both for ACS 2010, was low (r = -0.33) indicating that while lower income neighborhoods were more likely to have higher income inequality, the variables were measuring different aspects of economic status. Correlation between the NEI and median household income for ACS 2010 was 0.83 (p<0.001).

### HANDLS study population

The 3675 participants in HANDLS represent 60% African Americans and 40% Whites living in Baltimore City, Maryland with 59% living at or above poverty status and 41% below (Table 1). Participants ranged in age at enrollment from 30–64, with an average age of 48. Economic status of participants included those with high incomes; 20% of those answering the detailed questionnaire had a total annual household income of $50,000 or more (13% of African Americans and 29% of Whites). From enrollment (2004–2009) through 2013, 324 participants died. The most common cause of death was cardiovascular disease (N = 95) accounting for 29% of the total mortality, followed by cancer (N = 75, 23%). These were the two most common causes of death for both races, but Whites had almost equal numbers of cardiovascular disease and cancer deaths (28 and 30) while over half (N = 67, 62%) of the deaths in African Americans were due to cardiovascular disease (S2 Table). HIV/AIDS deaths primarily occurred in African Americans below poverty, but accounted for only 8% of the total deaths in the cohort.

**Table 1:** Characteristics of the Healthy Aging in Neighborhoods of Diversity Across the Life Span Study Participants, Baltimore, Maryland, 2004–2013 (N = 3675).

	African American		White	
Variable	Above Poverty	Below Poverty	Above Poverty	Below Poverty
Participants, no.	1156	1041	995	483
Men, %	47	44	48	40
Deaths, no. (%)	70 (6)	146 (14)	65 (7)	43 (9)
Age at enrollment, no. (%)				
30–34	119 (10)	112 (11)	106 (11)	53 (11)
35–39	140 (12)	135 (13)	126 (13)	58 (12)
40–44	149 (13)	149 (14)	137 (14)	70 (14)
45–49	203 (18)	213 (20)	170 (17)	96 (20)
50–54	185 (16)	188 (18)	168 (17)	79 (16)
55–59	207 (18)	138 (13)	153 (15)	71 (15)
60–64	153 (13)	106 (10)	135 (14)	56 (12)
Education at enrollment, no. (%)				
<9 years	43 (4)	74 (7)	62 (6)	73 (15)
9–11 years	249 (22)	367 (35)	205 (21)	154 (32)
High School / GED	431 (37)	373 (36)	283 (28)	138 (29)
Some College	321 (28)	192 (18)	201 (20)	66 (14)
College Degree	104 (9)	30 (3)	189 (19)	24 (5)
Missing	8	5	55	28
Neighborhood Economic Index Score, mean (sd)	-1.1 (3.8)	-3.2 (4.9)	0.7 (3.2)	-0.6 (3.0)
Neighborhood Median Income, median	$32,214	$30,239	$36,957	$35,200
Gini Coefficient, mean (sd)	44 (6)	47 (7)	43 (6)	41 (5)

BMI: Body mass index calculated as kg/m^2^, missing for 867 participants

Neighborhood Economic Index based on American Community Survey 5-year Estimate Data, 2006–2010

Gini Income Inequality Coefficient from American Community Survey 5-year Estimates, 2006–2010, multiplied by 100

At their initial visit, HANDLS participants lived in 46 unique census tracts within the city limits of Baltimore, Maryland (S1 Fig). These 46 tracts had a mean NEI of -0.79 (standard deviation = 4.6) and were not significantly different in NEI value than the entire 198 tracts of Baltimore (t-test, p = 0.36). The included tracts had a neighborhood median income ranging from $12,384 to $87,619 compared with Baltimore overall which ranged from $9412 to $133,548. The 46 tracts had a median income level of $32,738 and the distribution was slightly positively skewed (skewness = 1.04). The average NEI for all HANDLS participants was -1.13 (median = -1.31, standard deviation = 4.2), with African American participants generally living in neighborhoods with lower NEI values than Whites (t-test, p<0.001). The average Gini coefficient was 44 (median = 43, standard deviation = 6.5). NEI values had low correlations with individual socioeconomic variables of poverty status (r = -0.33) and education level (r = 0.19).

There were 324 deaths among the 3675 participants between enrollment and the end of 2013. Participants were followed for 6.9 years on average for a total of 25,186 person-years. Model selection using NEI (Model 1) or neighborhood median income (Model 2) resulted in the same general model structure (Table 2). The Cox regression models for overall mortality identified a significant three-way interaction among sex, race and poverty status with African American men living below poverty having the lowest survival and African American women living above poverty the highest. There was a differential mortality risk between African Americans and Whites across sex and individual poverty status (Table 3). African American men living below poverty had almost twice the mortality risk as their White counterparts (Model 1: HR 1.95; CI: 1.09, 3.51 and Model 2: HR 1.99; CI: 1.11, 3.57). The Gini coefficient was significantly related to overall mortality in an interaction with poverty status, although the combined association was small. High Gini values were associated with higher HR for poverty status on mortality than those for low Gini values. For African American men, those below poverty had more than twice the mortality risk as those above poverty at the 75th percentile of income inequality, but the increased risk for those below poverty was only 81% higher at the 25th percentile of income inequality (Model 1, high Gini values: HR: 2.37; CI: 1.60, 3.52 and low Gini values: HR 1.81, CI: 1.14, 2.89).

**Table 2:** Multivariable Cox Regression Analysis on Overall Mortality, Healthy Aging in Neighborhoods of Diversity across the Life Span Study, Baltimore, Maryland, 2004–2013 (N = 3675).

Variable	Model 1 HR	95% CI	Model 2 HR	95% CI
Sex				
Male	1.51	0.92, 2.47	1.51	0.92, 2.48
Female (ref)	1.00		1.00	
Race				
African American	0.86	0.51, 1.44	0.83	0.49, 1.40
White (ref)	1.00		1.00	
Poverty Status				
Above (ref)	1.00		1.00	
Below	0.42	0.09, 1.95	0.37	0.08, 1.72
Gini Coefficient	0.98	0.96, 1.01	0.98	0.95, 1.00
NEI	0.96	0.93, 0.98*	--	
Neighborhood Median Income**	--		0.84	0.75, 0.95*
Sex × Race	0.97	0.49, 1.93	0.98	0.49, 1.93
Sex × Poverty	0.47	0.21, 1.06	0.47	0.21, 1.06
Race × Poverty	0.76	0.38, 1.54	0.81	0.40, 1.63
Poverty × Gini Coefficient	1.04	1.00, 1.07*	1.04	1.00, 1.07*
Sex × Race × Poverty	3.03	1.12, 8.19*	3.00	1.11, 8.11*

HR: Hazard ratio, CI: Confidence Interval, NEI: Neighborhood Economic Index

* p<0.05

**Neighborhood median income in units of 10,000 (e.g., 1 = $10,000)

**Table 3:** Mortality Hazard Ratios and 95% Confidence Intervals for African Americans relative to Whites by Sex and Poverty Status, Healthy Aging in Neighborhoods of Diversity across the Life Span Study, Baltimore, Maryland, 2004–2013 (N = 3675).

	Below Poverty		Above Poverty	
	HR	95% CI	HR	95% CI
**Model 1: Neighborhood Economic Index**				
Male	1.95	1.09, 3.51	0.84	0.53, 1.33
Female	0.66	0.41, 1.06	0.86	0.51, 1.44
**Model 2: Neighborhood median income**				
Male	1.99	1.11, 3.57	0.81	0.51, 1.29
Female	0.68	0.42, 1.08	0.83	0.49, 1.40

HR: Hazard ratio, CI: Confidence interval

Models included three-way interaction of sex, race and individual poverty status and two-way interaction of individual poverty status and neighborhood Gini coefficient

Both NEI and median household income were significant when added to models separately. NEI was significantly and negatively associated with overall mortality, indicating that participants in areas of higher economic level had a lower risk and thus greater survival than their counterparts living in lower economic areas. The 75th percentile of NEI values for HANDLS participants had a 20% decrease in mortality relative to those at the 25th percentile of NEI values (HR: 0.80, CI: 0.69, 0.91). Neighborhood median income, in units of $10,000, was significantly and negatively associated with overall mortality. The 75th percentile of median household income values for HANDLS participants had a 17% decrease in mortality relative to those at the 25th percentile of median household income values (HR: 0.83, CI: 0.73, 0.94). Both models had adequate fit upon testing and review of the Schoenfeld residuals. The AIC scores for the NEI Model 1 of 4137.5 and neighborhood median household income Model 2 of 4139.0 indicate that the models performed similarly.

## Discussion

This study uses the power of the HANDLS stratified sample to examine race, and individual and neighborhood economic status as they relate to disparate rates in mortality. We found that overall neighborhood economic status and income inequality for those below poverty were independently related to mortality, beyond the synergistic effects of sex, race and individual poverty status. While African American men living below poverty had the highest overall mortality among the sex, race and individual poverty groups, higher levels of neighborhood economic status were associated with decreased mortality for all. This effect held whether NEI or neighborhood median household income was used as the measure of neighborhood economic status. We showed that the NEI was an objective measure of neighborhood economic status with high internal validity, consistency over short time periods, and low redundancy with individual measures of socioeconomic status, although neighborhood median household income had the same relationship with mortality and resulted in a similar model.

While individual factors of race, sex and individual poverty level are known to be related to mortality, we identified a significant interaction among these variables. African American men with household incomes below 125% of the federal poverty level had the highest risk of mortality compared to other race, sex and poverty groups in the study. Racial disparities in mortality have persisted over the last century [37], and disparities due to poverty [38] and gender [39, 40] are persistent and profound. African American men have a lower life expectancy (71.8 years) than White men (76.5), but the synergistic association of these three variables in HANDLS with mortality suggest that several social conditions associated with health may unequally affect African American men in poverty.

African American men may experience exceptional barriers to maintaining their health in their communities. African Americans in prison have lower mortality than non-institutionalized men; African American male prisoners aged 15–64 have an age adjusted mortality rate 43% lower than the general population [41]. Prison has a protective effect against the leading causes of death that differentially impact non-institutionalized African American men. Prison may also provide access to continuous adequate healthcare that manages existing conditions and treats any new diseases that emerge during the term of confinement. While prison is an unhealthy environment associated with greater mortality for women and for White men, prison mitigates the disproportionate mortality rate suffered by African American men when residing outside the prison walls. Gains in life expectancy and improvement in health status during the last 60 years that resulted from governmental social and economic policies improved the health of African Americans; however, African American men did not benefit as much as African American females [42, 43].

Governmental economic policies that improved employment and income for African Americans also differentially benefitted African American women, leaving many African American men behind [44]. Unemployment in African American men is twice the rate for White men in the US [45], and is associated with increased mortality [46]. Although the education gap is narrowing nationally between African Americans and Whites, African Americans continue to have lower standardized reading and math scores [47]. In Baltimore, African American 4th grade students scored lower than Whites in reading assessments, males score lower than females, and students qualifying for free/reduced lunch scored lower than those not eligible [48]. For 2013–2014, the estimated 4 year national high school graduation rate for African American males was 59% compared to 80% from white males [49]. In Maryland, the gap between African American males and White males was 17%. Graduation rate disparities for African American males exist at the undergraduate level and particularly at the graduate level exemplified best by the 36 year stagnation in application and matriculation rates of African American males in medical school [50]. Educational attainment's relationship with mortality has changed over time, but they continue to be strongly associated [51].

Racial and economic disparities are often confounded, which along with residential segregation yields racial and spatial differences in health [7]. In HANDLS, as in other studies of metropolitan areas [52], African Americans were more likely to live in areas of lower economic status regardless of their individual economic status. In the US overall, income segregation among African Americans families is 60% greater than among White families [9]. The greater Baltimore-Towson metropolitan area ranks 18th out of 117 metropolitan areas in the US in family income segregation with 29% of the families living in either poor or affluent neighborhoods. Baltimore has a long history of residential segregation by race. In 1911, Baltimore Mayor Mahool signed a segregation law separating city blocks for use by African Americans and Whites. Baltimore was one of the 239 urban areas with official 'residential security maps' used by the US Federal Housing Administration and private lenders during 1934–1968 to identify areas risky for mortgages, usually African American neighborhoods [53]. While the 1968 Federal Housing Law made discriminatory practices by lenders illegal, there is evidence that the practices continue. The cities of Baltimore, MD and Washington, DC reached a settlement with Wells Fargo regarding steering approximately 4000 African American and Hispanic borrowers during 2004–2008 into subprime mortgages when non-Hispanic White borrowers with similar credit profiles received prime rate loans [54]. The original redlined areas east and west of downtown Baltimore [55] are some of the areas with the lowest NEI using 2006–2010 ACS data (S1B Fig).

We identified a significant relationship between the NEI and mortality after accounting for individual level variables of race, sex and poverty status. This finding is similar to the association between neighborhood economic status and mortality observed in a recent larger study [13], although the HANDLS cohort includes a broader range of economic levels for both races, including 20% of those answering the questionnaire having an annual household income greater than $50,000. The health impact of neighborhood economic status may be through differences in access to healthy foods [56, 57], exposure to crime and stress [58], and differences in access to health care [59], or other less well-established factors such as proximity to sources of toxic pollutants [60], inadequate city services such as infrequent trash disposal, or lax hygienic enforcement leading to rodent infestations. A study of census tracts in Alameda County, California identified an interaction between neighborhood economic status and individual income level on mortality [25]. Low-income individuals had the highest mortality risk in the highest neighborhood economic status level. It could be that our inclusion of neighborhood income inequality accounted for a possible interaction between these variables in the HANDLS cohort.

The association between income inequality and mortality differed based on individual poverty status. High levels of income inequality were associated with higher HR for poverty status on mortality than for those with low levels of income inequality. There are well-known effects of macro-level income inequality on health. A recent review concluded that large income disparities damage health, and that countrywide income disparities are increasing over time [61]. In ecological studies in the US, the association of income inequality and mortality differs by the racial composition of the area considered [62, 63], and the degree of racial segregation confounds the income inequality/mortality relationship among African Americans [64]. Poor African American families have a higher degree of segregation in US urban areas than other poor racial groups [65], which may lead to confounding in ecological studies. There are fewer reports of the effects of micro-level inequality, and fewer still of the association of income equality and individual-level differences in survival. Research reviews support an overall significant negative effect of inequality on health [21, 23], however, the results may depend on the spatial aggregation considered [66] and whether perceived health or actual health outcomes are used. Researchers have found an increased likelihood of coronary heart disease [67] and obesity [21] for those living in areas with greater income inequality. These findings correspond with the current study where the most common cause of death was cardiovascular disease.

This study has several limitations. The HANDLS sample is representative of the diverse urban-dwelling population in Baltimore, Maryland, and may not be representative of African Americans and Whites living in other areas, especially those in suburban or rural communities. Independent demographic analyses of the HANDLS sample determined it representative of urban populations from U.S. cities with similar population densities and racial distribution, namely, Atlanta, GA; Bridgeport, CT; Bridgeton, NJ, Buffalo, NY; Camden, NJ; Carson, CA; Chicago, IL; Cleveland, OH; Detroit, MI; Harrisburg, PA; Hartford, CT; Oakland, CA; Springfield, MS; and Trenton, NJ [68]. Also, several variables that may have further explained the results, such as incarceration history or wealth, were not collected in the HANDLS study. The neighborhood level information was compiled at the census tract level and may not represent meaningful neighborhood units. However, census tract analysis has been as consistent as smaller census blocks, and more sensitive to gradients and change than larger zip code groupings [17]. Finally, only neighborhood data at study enrollment was included. Participants may have moved to better or worse neighborhoods during the follow-up period. The primary strength of this study is the HANDLS design which includes people above and below poverty for both races living in the same city.

Use of composite indices has been a natural solution to measuring the complex social and economic factors in a small area which could affect health outcomes. While originally these indices were based on previous research and theory [69], recently indices have been developed more objectively using analytic approaches such as principal component analysis [16]. We introduced the NEI as an empirically derived measure of neighborhood economic status separate from racial neighborhood composition. For HANDLS, the NEI was consistent over two time periods and had high internal reliability. The Cox model with the NEI was similar to that using the median household income, supporting the results by Oka [19] who found that the variation in neighborhood affluence-deprivation across urban cities could be accounted for by median household income as well as by a composite index. We extend these findings by demonstrating that neighborhood median income provided the same explanatory power as an objectively derived index in terms of mortality for the HANDLS study.

This study leveraged the HANDLS study's unique factorial design of race, sex, age and individual poverty status, measuring time to death as an objective measure of health, and included both neighborhood economic status and income inequality for a population of middle-aged urban-dwelling adults. Our findings add to the current body of knowledge by describing the combined association of race, sex and individual poverty with mortality in an adult cohort. African American men living below 125% of the federal poverty level were disproportionately likely to suffer early mortality. The additional neighborhood variables of economic status, as measured by the NEI, and income inequality, as measured by the Gini coefficient significantly added to the model, indicating the separate association of these variables with mortality.

Future research should examine in more detail the effect of neighborhood economic level on mortality by taking into account movement of people over time, as well as examining possible interventions. While our findings support the use of median household income across small areas as an indicator of overall neighborhood economic status, it should be explored if these findings hold for suburban and rural environments.

## Supporting Information

S1 Appendix Neighborhood Economic Index Development. (DOC)

S1 Fig Locations of Healthy Aging in Neighborhoods of Diversity Across the Life Span African American and White Participants in Baltimore, Maryland 2004–2009, with Gini Income Inequality Coefficient (A) and Neighborhood Economic Index (B) by Census Tract.
(PDF)

S1 Table Census Tract Variables and Principal Component Loadings for Inclusion in the Neighborhood Economic Index, Baltimore, Maryland. (DOC)

S2 Table Detailed characteristics of Deaths to Participants in the Healthy Aging in Neighborhoods of Diversity Across the Life Span Study, Baltimore, Maryland, 2004–2013 (N = 3675). (DOCX)
