# Supplementary material for: Race, Neighborhood Economic Status, Income Inequality and Mortality
Source: PLoS One. 2016 May 12;11(5):e0154535. doi: 10.1371/journal.pone.0154535 (PMC4865101; doi:10.1371/journal.pone.0154535)
Supplement: S1 Appendix — (DOC) [file pone.0154535.s001.doc]

S1 Appendix: Neighborhood Economic Index Development

METHODS

The 19 selected neighborhood-level variables from the American Community Survey (ACS) 5-year estimate files were examined for skewness, and all variables were standardized by subtracting the mean and dividing by the standard deviation. The standardized variables were included in a principal component analysis (PCA) and bootstrapping was used to estimate the 95% confidence intervals (CIs). Variables that had absolute loadings with CIs greater than the median loading were retained for the index. Retained standardized variables were summed to create the index value. Repeatability of the method was assessed by performing the variable selection method on the ACS 2013 dataset. Correlations of the index values calculated for the ACS 2010 and 2013 datasets indicate the degree of consistency over time.

RESULTS

Baltimore, Maryland has 200 census tracts and 198 of them contain at least one household. None of the 19 variables considered in the ACS 2010 data had an absolute skewness value greater than 2, and all were standardized to a mean of 0 and standard deviation of 1. PCA of the standardized variables resulted in a primary component accounting for 45% of the variance. The next component accounted for only 14% of the variance, and thus only the primary component was retained. The loadings on the primary component ranged in absolute value from 0.006 to 0.300 (S1 Table), with a median absolute loading of 0.230. Six of the variables met the condition of having a 95% CI greater than the median loading: percent of households with unemployed, percent of households with people out of the workforce, percent of households receiving food stamps, percent of households earning less than $30,000 annually, percent of households with no car and percent of households in poverty. NEI values were calculated as the sum of the six individually standardized variables, and varied from -16.6 to 10.3, with a median value of 0.5 (mean=0).

To examine the repeatability of the method, the variable selection process was conducted on ACS 2013 data for the 198 census tracts. Again, none of the 19 variables considered had an absolute skewness value greater than 2, and all were standardized. PCA of the standardized variables resulted in a primary component accounting for 47% of the variance, with the second component accounting for only 17%. The loadings on the primary variable varied in absolute value from 0.008 to 0.301 (S1 Table), with a median absolute loading of 0.214. The six variables identified in the ACS 2010 dataset were also identified in the ACS 2013 dataset along with two others (percent of households receiving supplemental social security, and percent of households with adults having less than a high school education). These two additional variables had the lowest loadings of the identified variables. The consistency of the six original variables identified in the ACS 2010 dataset supported their use in NEI index. NEI values based on the ACS 2013 dataset varied from -14.8 to 10.7, with a median value of 0.16 (mean=0).

The correlation between the NEI for ACS 2010 and ACS 2013 was 0.96 (p<0.001) indicating high agreement over time.
