# Supplementary figures and images for: Race, Neighborhood Economic Status, Income Inequality and Mortality
Source: PLoS One. 2016 May 12;11(5):e0154535. doi: 10.1371/journal.pone.0154535 (PMC4865101; doi:10.1371/journal.pone.0154535)

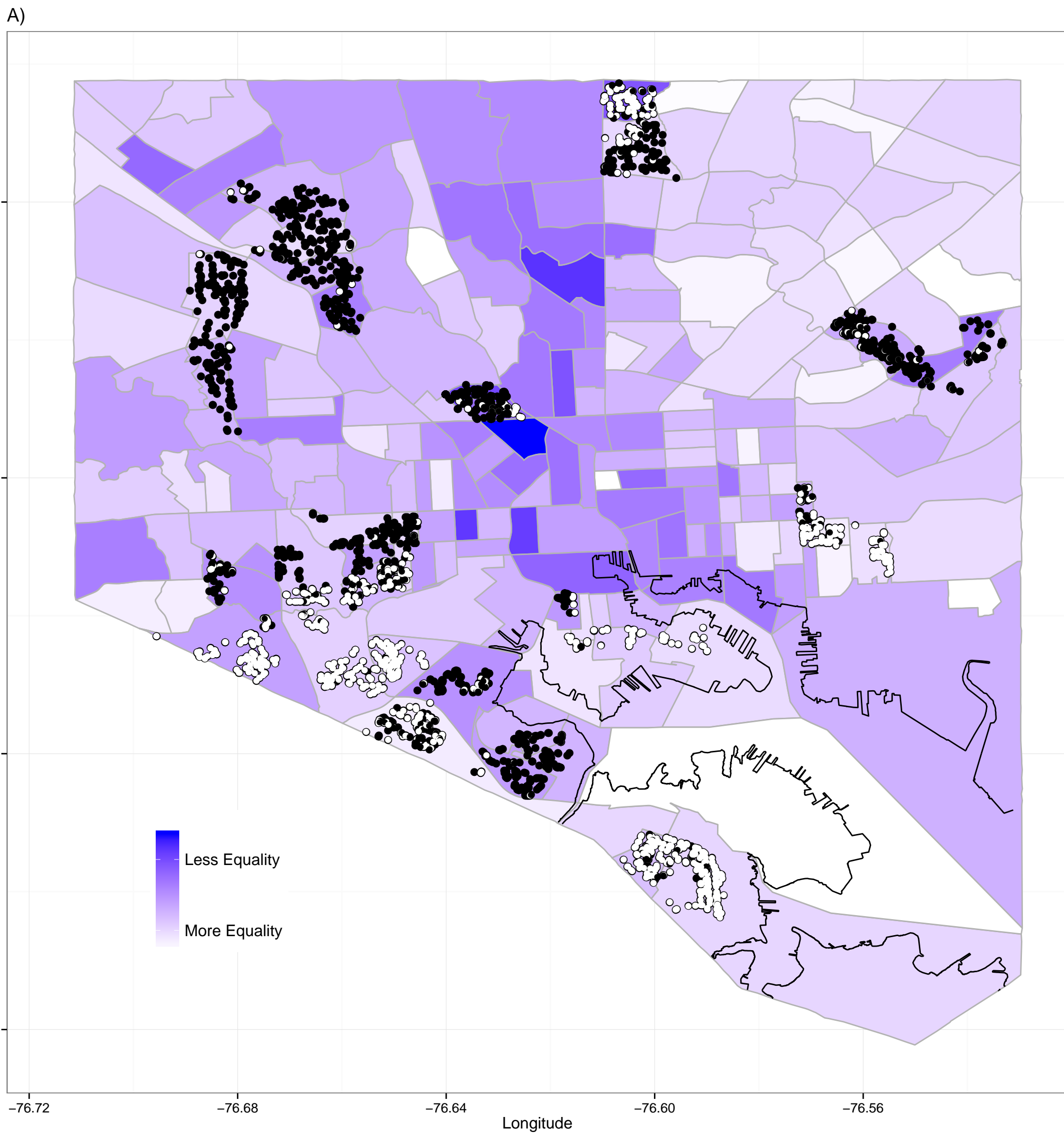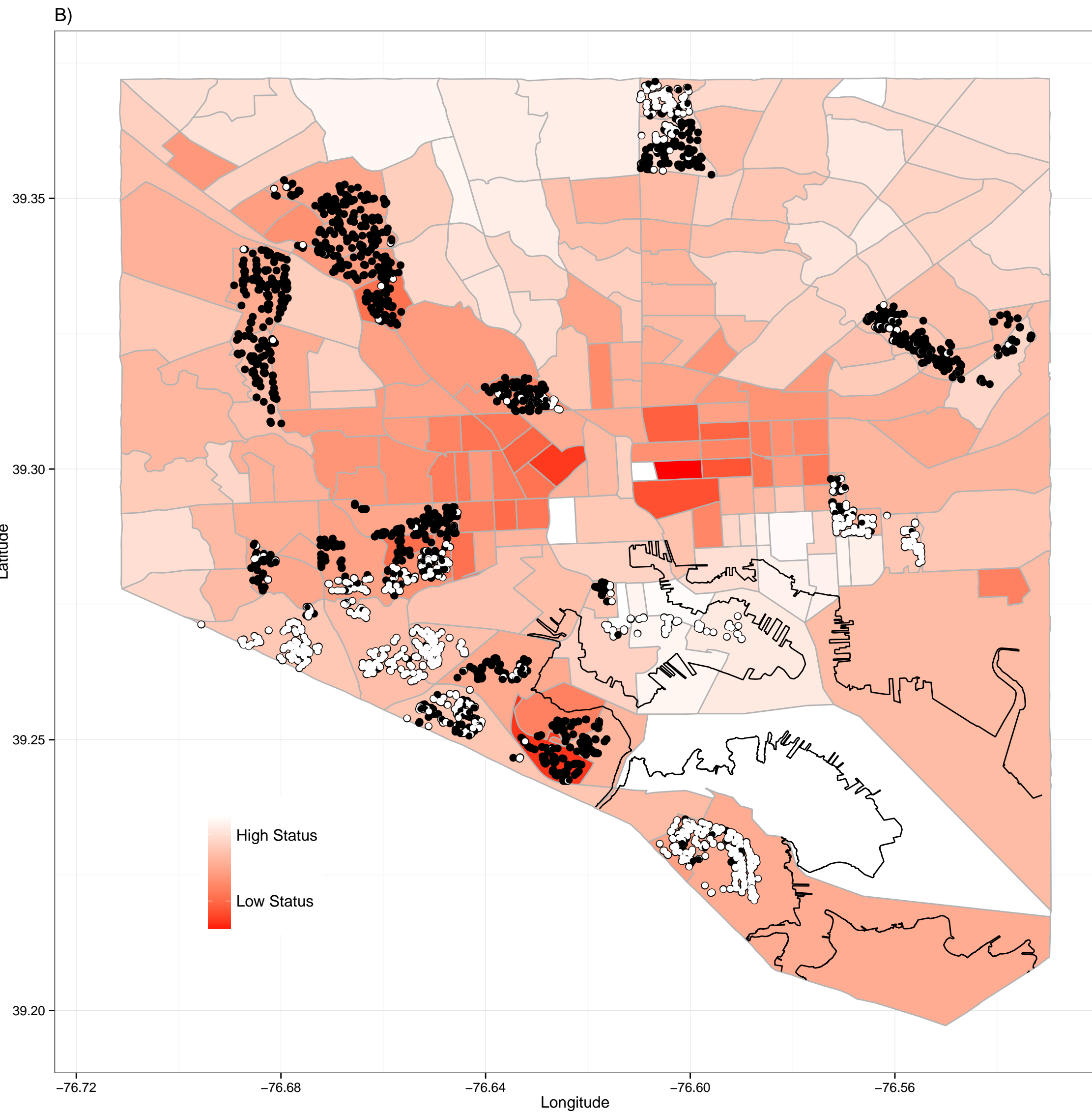

Supplement: S1 Fig — Locations of Healthy Aging in Neighborhoods of Diversity Across the Life Span African American and White Participants in Baltimore, Maryland 2004–2009, with Gini Income Inequality Coefficient (A) and Neighborhood Economic Index (B) by Census Tract. (PDF) [file pone.0154535.s002.pdf]
