# Supplementary material for: Race, Neighborhood Economic Status, Income Inequality and Mortality
Source: PLoS One. 2016 May 12;11(5):e0154535. doi: 10.1371/journal.pone.0154535 (PMC4865101; doi:10.1371/journal.pone.0154535)
Supplement: S1 Table — (DOC) [file pone.0154535.s003.doc]

**S1 Table. Census Tract Variables and Principal Component Loadings for Inclusion in the Neighborhood Economic Index, Baltimore, Maryland**

| **Variable** | **ACS 2010^*^** |  | **ACS 2013^†^** |  |
| --- | --- | --- | --- | --- |
|  | **Loading** | **95% CI*** | **Loading** | **95% CI*** |
| Percent adults with less than a high school education | 0.252 | 0.221, 0.281 | **0.265** | **0.239, 0.289** |
| Percent adults unemployed | **0.289** | **0.264, 0.310** | **0.284** | **0.261, 0.301** |
| Percent adults no longer in work force | **0.284** | **0.255, 0.309** | **0.272** | **0.240, 0.298** |
| Percent housing rented | 0.209 | 0.162, 0.249 | 0.207 | 0.165, 0.244 |
| Percent housing vacant | 0.202 | 0.153, 0.251 | 0.188 | 0.137, 0.241 |
| Percent housing crowded (more than one occupant per bedroom) | 0.129 | 0.074, 0.184 | 0.119 | 0.067, 0.170 |
| Percent males in management, business, science, and arts occupations | -0.227 | -0.269, -0.181 | -0.229 | -0.267, -0.189 |
| Percent females in management, business, science, and arts occupations | -0.234 | -0.276, -0.189 | -0.235 | -0.273, -0.193 |
| Percent households in poverty | **0.284** | **0.248, 0.314** | **0.286** | **0.257, 0.311** |
| Percent households earning under $30,000/year | **0.300** | **0.275, 0.320** | **0.298** | **0.276, 0.316** |
| Percent households receiving social security | 0.122 | 0.061, 0.184 | 0.100 | 0.042, 0.155 |
| Percent households receiving supplemental social security | 0.251 | 0.214, 0.285 | **0.274** | **0.245, 0.300** |
| Percent households receiving food stamps | **0.298** | **0.271, 0.320** | **0.301** | **0.282, 0.317** |
| Percent households on public assistance | 0.225 | 0.180, 0.275 | 0.221 | 0.178, 0.265 |
| Percent households with no car | **0.300** | **0.277, 0.318** | **0.296** | **0.273, 0.314** |
| Percent households in same residence since 2005 | -0.019 | -0.082, 0.039 | -0.008 | -0.066, 0.053 |
| Percent female headed households with dependent children | 0.244 | 0.206, 0.275 | 0.235 | 0.196, 0.268 |
| Percent renter or owner costs in excess of 50% of income | 0.167 | 0.110, 0.219 | 0.192 | 0.208, 0.252 |
| Percent adults 65 years or older | -0.006 | -0.078, 0.062 | -0.016 | -0.073, -0.026 |

* American Community Survey 5-year Estimate File 2006-2010

** Principal component analysis loading on primary factor, 95% confidence intervals based on bootstrapped values, bold values indicate absolute intervals greater than the absolute median loading value (0.230 for 2010, 0.214 for 2013)

† American Community Survey 5-year Estimate File 2009-2013
