# Supplementary material for: Race, Neighborhood Economic Status, Income Inequality and Mortality
Source: PLoS One. 2016 May 12;11(5):e0154535. doi: 10.1371/journal.pone.0154535 (PMC4865101; doi:10.1371/journal.pone.0154535)
Supplement: S2 Table — (DOCX) [file pone.0154535.s004.docx]

**S2 Table. Detailed characteristics of Deaths to Participants in the Healthy Aging in Neighborhoods of Diversity Across the Life Span Study, Baltimore, Maryland, 2004-2013 (N=3675)**

|  | **African American** | | **White** | |
| --- | --- | --- | --- | --- |
| **Variable** | **Above Poverty** | **Below Poverty** | **Above Poverty** | **Below Poverty** |
| Participants | 1156 | 1041 | 995 | 483 |
| Deaths | 70 | 146 | 65 | 43 |
| Gini Coefficient |  |  |  |  |
| Gini Percent ≤ 43.1 (%) | 27 (5) | 23 (8) | 52 (8) | 34 (8) |
| Gini Percent > 43.1 (%) | 43 (7) | 123 (16) | 13 (4) | 9 (11) |
| Neighborhood Economic Index (NEI) |  |  |  |  |
| NEI ≤ -1.31 (%) | 44 (7) | 115 (16) | 33 (10) | 19 (8) |
| NEI > -1.31 (%) | 26 (5) | 31 (10) | 32 (5) | 24 (10) |
| Primary Causes of Death |  |  |  |  |
| Cardiovascular Disease (%) | 28 (40) | 39 (27) | 15 (23) | 13 (30) |
| Cancer (%) | 12 (17) | 33 (23) | 21 (32) | 9 (21) |
| HIV/AIDS (%) | 4 (6) | 21 (14) | 1 (2) | 0 |
| Other (%) | 26 (37) | 53 (36) | 28 (43) | 21 (49) |

HIV/AIDS: Human immunodeficiency virus, Acquired immunodeficiency syndrome (ICD 10 B20-B24); Cardiovascular Disease (ICD10 I00-I9); Cancer (ICD10 C00-C97)

Poverty is 125% of the household US Federal Poverty Level
